# ECM-Based Materials in Cardiovascular Applications: Inherent Healing Potential and Augmentation of Native Regenerative Processes

**DOI:** 10.3390/ijms10104375

**Published:** 2009-11-20

**Authors:** Anna V. Piterina, Aidan J. Cloonan, Claire L. Meaney, Laura M. Davis, Anthony Callanan, Michael T. Walsh, Tim M. McGloughlin

**Affiliations:** Centre for Applied Biomedical Engineering Research (CABER), Department of Mechanical & Aeronautical Engineering, and Materials and Surface Science Institute (MSSI), University of Limerick, Limerick, Ireland; E-Mails: anna.piterina@ul.ie (A.V.P.); aidan.cloonan@ul.ie (A.J.C.); claire.meaney@ul.ie (C.L.M.); laura.davis@ul.ie (L.M.D.); anthony.callanan@ul.ie (A.C.); michael.walsh@ul.ie (M.T.W.)

**Keywords:** vascular graft, extracellular matrix, healing, native regenerative processes

## Abstract

The *in vivo* healing process of vascular grafts involves the interaction of many contributing factors. The ability of vascular grafts to provide an environment which allows successful accomplishment of this process is extremely difficult. Poor endothelisation, inflammation, infection, occlusion, thrombosis, hyperplasia and pseudoaneurysms are common issues with synthetic grafts *in vivo*. Advanced materials composed of decellularised extracellular matrices (ECM) have been shown to promote the healing process via modulation of the host immune response, resistance to bacterial infections, allowing re-innervation and reestablishing homeostasis in the healing region. The physiological balance within the newly developed vascular tissue is maintained via the recreation of correct biorheology and mechanotransduction factors including host immune response, infection control, homing and the attraction of progenitor cells and infiltration by host tissue. Here, we review the progress in this tissue engineering approach, the enhancement potential of ECM materials and future prospects to reach the clinical environment.

## 1. Introduction

Extensive multidisciplinary research, especially in the latter half of the 20th century (early 1950s), has focused on the replacement of damaged, occluded, ruptured and atherosclerotic blood vessels [1–3]. Candidate materials have evolved from inert gold tubular structures [2] to the advanced synthetic prosthetic grafts found in modern clinical settings [1,3,4]. Cardiovascular disease (CVD) is currently one of the leading causes of death in the Western world, hence vascular grafts with increasing requirements are being used to a greater degree and in a wider variety of clinical scenarios [1].

The molecular mechanisms involved in prosthetic vascular graft healing, as well as detailed analysis of healing kinetics in animal and human models are described in detail by Davis *et al.* [4] and Greisler *et al.* [5]. They revealed that the graft healing process represents an intricate, interconnected multicellular process which is the result of a variety of host body response mechanisms; however certain factors of the healing process are highly conservative and determines the total outcome for the restoration of vessel functionality. The graft healing process involves the coordination of host immune cells activity, migration, infiltration, proliferation and differentiation of endothelial cells (ECs), smooth muscle cells (SMCs) and their progenitors which culminate in the formation of new tissue. Their collaborative, balanced regulation and maintenance are pivotal for successful graft implantation. Disruption to the inter-regulation of this process or the negative effect of exogenous factors (bacterial infection, nutrition deficiencies, stresses and a variety of neurological factors) are frequent causes for failures of synthetic grafts by provoking pathological issues such as thrombosis, anastomotic hyperplasia and limited re-endothelisation. [2–9].

Many attempts have been made to increase the patency rates of vessel grafts. Improvements in manufacture, pre-treatment, modification of surface charge and topography, incorporation of therapeutic agents and bioactive coatings*, in vitro* addition of a cell monolayer [4,10–14], have all been shown to improve the early stage human healing processes, but have not demonstrated ideal outcomes in terms of long-term performance [4,7]. The general concept of material bioinertness (nontoxic and non-antigenic) [15,16] for vascular synthetic grafts has been unsuccessful in stimulating positive healing responses and improving the long-term patency of the vascular graft.

An important alternative to synthetic materials is the use of extracellular matrix biomaterials derived from *ex vivo* tissues [17,18]. To date, numerous animal trials have demonstrated the performance of extracellular matrix (ECM) material for vascular graft applications, highlighting excellent healing properties during implantation for a variety of blood vessels (aorta, artery, vein) [19–25]. At certain sites of implantation, the healed graft site exhibited a histological appearance similar to that of normal blood vessel with evidence of early capillary penetration and full endothelisation. There was no evidence of infection, intimal hyperplasia, aneurysmal dilation and the site contained a smooth muscle media and a dense fibrous connective tissue adventitia [22].

A thorough elucidation of ECM material characteristics and their influences on the healing process is critical to the understanding of graft pathology and may lead to greater uses of ECM for augmentation and the enhancement of vascular substitutes which are currently available. The main aim of this review is to describe and summarise factors known to date which have been shown to play a beneficial role in modulating cellular events in relation to the healing process. Disadvantages of ECM materials are described in this paper and methods which aim to improve the tissue-specificity, biomechanical properties and total performance of ECM grafts for a variety of cardiovascular applications are presented. This review is aimed at scientists of interdisciplinary fields and describes many unresolved fundamental aspects of vascular graft healing, thus highlighting areas of interest of future research and development.

## 2. Origin of ECM Material: Source, Preparation, Biochemical Properties, Storage and Commercial Availability

The common goal of all proposed and developed decellularisation methodologies is to efficiently remove all cellular and nuclear material while minimising any adverse effects on the composition, biological activity and mechanical integrity of the remaining ECM. Preservation of the structural components of the ECM allows the generated matrix to provide structural integrity and biomechanical strength for newly developed tissues and enable efficient reseeding [17,18]. A thorough review of the various decellularisation techniques has been previously compiled by Gilbert *et al.* [18]. Biochemical techniques of decellularisation include osmotic shock, solvent extraction, ionic and non-ionic detergents, acid/alkaline treatments and enzymatic digestion with DNase, RNase, lipase and proteases. Recently developed methods based on the application of periodic pressure for decellularisation of the skin have been reported in literature [26]. In addition to these methods, bacterial enzymes produced by *Micrococcus luteus* have been investigated for removal of cellular components and their potential in achieving a high quality decellularised matrices [27]. Protocols vary greatly, and are all known to have limitations [18].

Numerous organs and tissues from warm-blooded animals (bovine, sheep, monkeys, pigs and rabbits), including humans, have been investigated to date as potential sources of ECM [18,28,29]. Tissues like the aorta [18], vein [30], bladder [18], small intestinal submucosa [18], skin [18], ureter [31], liver [18], amniotic membrane [32] and tendon [33] have been successfully decellularised and investigated.

ECM materials can be manufactured and obtained in multiple forms. Multi-laminate sheets can be prepared by vacuum pressing methods [18] and crosslinked by various chemical and enzymatic agents [34–37]. Particulate suspensions, powdered and gel forms of ECM can easily be prepared and delivered by minimally invasive techniques to the site of interest [38], where gel can self-assemble into 3D matrices. Material can be stored in the hydrated state without lyophilisation and cryopreserved [39]. Lyophilised forms of ECM have a long shelf life and are easily transportable. However, non-lyophilised, hydrated ECMs have shown excellent biomechanical and biochemical characteristics with improved cellular ingrowth rates, illustrating the importance of hydration on properties such as biological effects, degradability, mechanical properties of the structural components (strength and loading response) and volumetric change [40].

Naturally derived materials, such as porcine small intestinal submucosa (SIS), urinary bladder matrix (UBM), collagen and fibrogen, offer many mechanical, chemical and biological advantages over synthetic materials, due to their origin and nature [17,18]. Their natural three-dimensional ultrastructure and diverse composition of structural and functional proteins including collagen, elastin, growth factors and proteoglycans contibute to overall advantageous properties as a graft material [17,41–43]. However, it must be acknowledged that ECM scaffolds consist of structural and functional molecules secreted by the resident cells of each tissue and organ from which they are prepared. Therefore, the specific composition and distribution of the ECM constituents will vary depending on the tissue source [17,41–43]. The biochemical composition of several materials were evaluated and presented by Badylak *et al.* [17]. In brief, SIS scaffold material has been the most extensively characterized of all ECM materials. It is made up of approximately 90% collagen, the majority of which being type I, with minor amounts of types III, IV, V and VI also present. SIS ECM also contains glycosaminoglycans (GAGs), including heparin, heparin sulfate, chondroitin sulfate and hyaluronic acid. The amount of GAGs present in the tissue after processing depends greatly on the decellurisation methods used [18]. Previous studies have shown presence of the molecules fibronectin and laminin [44,45] which are known to provide adhesion ligands for cells, as well as growth factors [46] in SIS materials. UBM also contains the same collagen types as SIS but with greater amounts of type III as well as the presence of type VII, which is a central component of the endothelial basement membrane, a distinguishing characteristic of this matrix. Limited research was carried out on characterization and comparison of the spacial distribution of structural domain and the molecular components within the diverse range of ECM matrices. Localization and 3-D positioning of structural and biochemical components within the ECM matrices may have effect on cell adhesion and their migration pattern within the graft. With the use of 3-D imaging fluorescent microscopy, optical sectioning techniques and monoclonal antibody development, further investigation into the parameters mentioned above is possible. This information will help indentify the additional aspects and significance of material structure in relation to the rate of tissue healing.

A partial list of ECM-based, commercially available products is given in Tables 1 and 2. Form, source and composition of intact ECMs commercially exploited to date are diverse. This makes them highly desirable for many clinical applications. SIS is the most extensively clinically used ECM and has been used to reconstruct various tissues such as the abdominal wall, urinary bladder wall, tendons, intestine wall tissue, urethra and ureter [47–53]. There are a variety of commercially available collagenous matrices, typically consisting of renatured-purified collagen lyophilised to form sheets or wafers. Examples include collagen type I sheets, collagen matrix (collagen type I and types I and III), connective tissue composites and collagenous gels. Two of the most established products in clinical use are Dermagraft® (Anginera™) and Transcyte®. These are most commonly used in the treatment of diabetic foot ulcers and surgically excised full-thickness and deep partial-thickness thermal burn wounds, respectively. Dermagraft® is a cryopreserved living allogenic dermal equivalent, made from neonatal foreskin fibroblasts cultured on a bioresorbable polyglycolic (Dexon®) or polyglactin (Vicryl®) polymer matrix, and characterised by production of growth factors such as vascular endothelial (VEGF). Transcyte® is a temporary skin cover made from a collagen-coated non-bioresorbable nylon mesh seeded with allogenic neonatal human dermal fibroblasts. Both products are now being investigated for off-label applications with promising results.

## 3. Factors and Molecular Mechanisms of the ECM Bioactivity during Healing

### 3.1. The Early Response and Healing of Vascular Graft Material

Following material implantation, one of most important determinants for the acceptance and healing process is the immune cascade [54–57]. Due to their surface properties, microstructure, degree of antigenicity of structural compounds, ability to absorb plasma proteins and overall porosity, biomaterials can directly influence the adhesion and differentiation processes of adaptive and innate immune cells. Macrophages are the dominant infiltrating cells which respond rapidly to biomaterial implantation in tissues [58,59]. These cells and their fused morphologic variants usually remain at biomaterial-tissue interfaces for the life-time of the device *in vivo*. As a component of the immune system, macrophage activities are closely related to immune responses, inflammation and foreign body responses. However, macrophages also mediate the biodegradation of bioresorbable materials via phagocytosis and extracellular degradation [60]. Macrophages are essential for the regeneration process which occurs via alteration of reactive oxygen intermediate production, cytokine secretion [55–57] and the regulation of recruitment, proliferation and differentiation of vascular cells [61].

**Figure 1.**
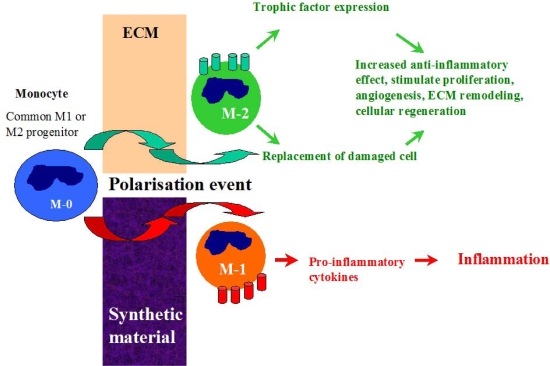
Pathways of monocyte differentiation during interaction with synthetic and ECM materials.

After graft implantation into the host, monocytes from the circulatory system are attracted to the site and may undergo distinctive pathways of differentiation (polarization) into either classically activated M-1 macrophages or the alternative M-2 phenotype [62]. This process is highly dependent on the graft material properties [55,57,63]. During interaction with synthetic graft materials, monocytes activate M-1 inflammatory pathways and release pro-inflammatory cytokines which propagate into inflammation and fibrosis (Figure 1). In contrast, the dynamic process of macrophage infiltration in ECM material has been shown to stimulate polarization of monocytes into the M-2 anti-inflammatory phenotype which is generally characterised by enhanced secretion of regenerative trophic factors which promote cell proliferation, reduce apoptosis and stimulate angiogenesis, and is considered to be a ’healing’ macrophage type [63]. The M-2 macrophages also have a capacity to transdifferentiate into ECs, provide local replacement of damaged cells and enchance endothelisation rate of the graft surface [64]. The presence of the M-2 macrophage type and its ability to secrete cytokines with chemotactic and mitogenic factors while simultaneously providing partial degradation of the ECM material in order to facilitate new tissue in-growth, are all factors which have beneficial effects on the early stage of healing.

Patients implanted with vascular grafts exhibit T-cell activation which is biomaterial induced [56,57,63], and leukocyte Th1/Th2 response has previously being characterized for SIS-ECM material [63]. Characterisation of tissue cytokines showed that SIS strongly stimulated the expression of IL-4 and development of Th2 phenotype, while the expression of IFN-γ (Th1) was 100 fold less than the response elicited by the xenogeneic biomaterial. It was concluded that the source of ECM did not alter the restricted Th2 immune response [62,65]. The Th2 response elicited by SIS does not adversely affect the hosts’ ability to mount a protective systemic immune response to T-dependent or T-independent vaccines or overcome viral or bacterial infections [66].

The Th2 response of macrophages during the integration ECM material may be partially explained by the lower hydrophobicity of the ECM surface compared with some synthetic materials [42]. Brodberk *et al.* [67] determined that leukocyte cytokine mRNA responses to implanted biomaterials are highly dependent on surface chemistry. Surfaces displaying various chemistries (hydrophobic, hydrophilic, anionic, and cationic) were placed into stainless steel cages and implanted subcutaneously. Semi-quantitative RT-PCR analyses revealed that hydrophilic surfaces showed a decreased expression of pro-inflammatory cytokines, IL-6 and IL-8.

ECM materials can locally modulate the host immune response via monocyte polarization (M2) or leukocyte activation (Th2) can suppress aggressive immune responses with no side effect or depletion of an immune reponse to bacterial infection or strong antigenic compounds, and can have a therapeutic effect and promote tissue remodeling.

### 3.2. Antimicrobial Resistance of ECM Materials

Vascular graft infection remains a serious complication with the application of synthetic graft materials [68–76]. The infection rate is highest for infrainguinal grafts at a rate of 2–5%, for aortofemoral grafts the rate is 1–2%, and for aortic grafts it is approximately 1%. Infection rates for prosthetic vascular graft infection range between 1–6%, between 0.5–5% for aortic graft infections [73–75] and 4–20% for prosthetic arteriovenous (AV) access grafts [76].

The most common organisms which are known to be causative agents of graft infections belong to various bacterial species: Gram-positive bacteria (such as *Staphylococcus aureus, Staphylococcus epidermidis, Streptococcus viridans,* and *Streptococcus faecalis*), and the Gram-negative bacteria species (*Escherichia coli, Pseudomonas aeruginosa, and Proteus mirabilis*). Anaerobes (*Clostridium sp*.) are rarely associated with the onset of infection, however they have been reported in a number of clinical cases in relation to aortic graft infection [77,78]. Cases of mycotic infection (*Candida sp*.) at graft sites have also been reported in literature [79].

Attachment of bacteria to the material surface is thought to be facilitated by numerous molecular mechanisms [80,81], which physical nature and strength are differ. For example, hydrophobic interaction [81], slime-mediated attachment and affinity attachment via specific–ligand binding to exposed chemical groups on the material surfaces [82–88].

The natural ability of microorganisms to adapt and apply various molecular mechanisms in order to adhere and develop a biofilm, make the concept of altering surface properties by physical, chemical and biological pre-treatment and modification techniques in order to provide an improved graft with minimal risk of infection, becomes much more difficult and complex.

The post-implantation healing process of vascular grafts is greatly affected and delayed by the presence of bacterial infection [89]. Bacteria are able to proliferate rapidly forming a highly protective microenvironment (mature biofilm) following initial attachment [90]. The hosts’ ability to control the removal and inactivation of these organisms is reduced as the biofilm matures and encapsulates in the “coat” composed with exopolysaccharides (EPS) [91]. The effectiveness of antibacterial drug penetration through the thick and chemically complex barrier is highly decreased [92–96]. This can have an adverse affect on the healing process through the production of destructive enzymes and endotoxins which have the ability to modulate and promote chronic inflammation [97–101]. Inflammatory response involving neutrophils and macrophages result in the release of free radicals [99] and numerous lytic enzymes (MMPs, TIMPs) [102] which can have a detrimental effect on the cellular processes involved in healing and can also cause damage to the surrounding tissue. Released proteases can affect growth factor activity, inhibit the process of accommodation of progenitor cells and/or the migration of fully differentiated vascular cells from near regions of the vessel and overall greatly affect tissue healing.

The limited success of pharmacological treatments for infected grafts continues to challenge vascular medicine. The process of infected graft replacement with a new one poses a high risk of re-infection at the site post-implantation and this is a major threat [68,103,104]. In order to prevent proliferation of the attached bacteria and inhibit the development of later stage infection, numerous approaches have been attempted to develop an infection-resistant graft with local delivery of the antibacterial component. For example, the incorporation of antibiotics [105–120] and silver nanoparticles [121,122] in vascular grafts material are commonly used approches for this purpose. However, these attempts provide only temporary solutions [123,124]. As we become more adept at managing the infections, the bacterial phenotype evolves into evermore virulent strains via mobilization of antibiotic–resistant genetic elements which become resistant to even more advanced antimicrobial agents [125]. In the case of silver application, lysed bacteria and absorbed protein on the surface of the coated material will increase the distance between the active coating layer and the microorganisms, this may lead to inefficiency of the antibacterial coating.

Recent studies have shown potent bacteriostatic activity of peptides which are obtained by acid/heat digestion of ECM material from various tissues and organs (liver, bladder, intestine) [126,127]. Activity against microorganisms was found to be of bacteriostatic nature (inhibit or delayed a proliferation process) and was determined by the presence of multiple peptides in the ECM. In this study, proliferation of Gram-positive and Gram-negative bacteria was shown to be inhibited for up to 12 hours by the peptide mixtures. This short-term effect was proposed to be beneficial in preventing the initial increase in bacterial number during initial healing and allowed the host inflammatory and humoral responses to be modulated. In contrast to synthetic graft material, during animal clinical studies processed ECM materials have shown to be resistant to persistent bacterial infection even after deliberate contamination with *Staphylococcus aureus* at the implantation site [128,129].

The origin of these antibacterial ECM peptides, the amino acid composition, degree of structural homology in relation to natural antimicrobial peptides (AMP) [130–133] and defensins [134], the pathways of their incorporation, storage and release *in vivo* from the ECM, are all aspects which have not been identified. The full spectrum of activity of low-molecular peptides has yet to be determined. A detailed evaluation of the antibacterial therapeutic properties of ECM materials needs to be fully investigated.

## 4. Vascular Tissue Development

### 4.1. Therapeutic Properties of the Remodeling Products of ECM Material

Endothelisation of the vascular graft is an important step during graft healing. ECs provide the optimal naturally occurring anti-thrombogenic and blood-compatible surface [8]. The development of an endothelial monolayer on the luminal surface of synthetic vascular graft, would prevent thrombus formation by inhibiting platelet accumulation on the graft surface [2–4], and hence improve long-term patency rates. There are currently three major mechanisms proposed for spontaneous *in vivo* endothelisation of vascular grafts: 1) the migration of ECs inward across the anastomosis from the native vessel (pannus ingrowth); 2) the deposition of circulating endothelial progenitor cells (EPCs) onto the luminal surface of synthetic vessel grafts; and 3) EC coverage derived from the ingrowth of capillaries through porous grafts (transmural endothelisation) [4,5,8,9].

Mature ECs originating from surrounding host vascular tissue may contribute to endothelial reconstruction on the graft by migrating to the material surface. However, mature ECs are terminally differentiated cells with a low proliferative potential, therefore their capacity to substitute damaged endothelium is limited. EPCs are a more valuable source of vascular endothelail cells and their effective accommodation at the graft site is an important requirement for vascular healing and tissue reconstruction [135–138]. The anatomical origin of these cells is an important factor which influences the rate of healing progress. Circulating EPCs, derived from peripheral blood, may give rise to fully matured, differentiated ECs when incorporated within injured vessels which then contribute to re-endothelisation and neo-vascularisation processes at the vascular graft [138]. The recruitment of EPCs from the bone marrow to homing sites of vasculogenesis is regulated by many factors, including chemokines and growth factors. The precise mechanism of EPC mobilization and differentiation is not entirely elucidated and is still under investigation [136]. There are factors which may inhibit the attachment of EPCs to the site of implantation [138]. The material properties and surface structure of the graft may not always provide a favorable microenvironment for their recruitment and may inhibit the rate of attachment and proliferation of EPCs [137,139]. Certain therapeutic compounds in patients with severe CVD lead to a reduction in the quantity and quality of circulating EPCs and greatly impacts the rate of the endothelium repair [140]. Graft implantation in these patients resulted in impaired healing and failure of the vascular graft due to dysfunctional endothelium.

Multiple *in vivo* clinical studies reveal that prosthetic vascular grafts often remain mostly without an endothelium, even after decades of implantation [141,142]. Several techniques have been proposed in order to improve synthetic graft performance [143]. The first option involves EPC therapy (exogenous introduction of vascular progenitors) in order to facilitate restoration and assist the healing of vascular grafts. This method has great potential for improving the quality of life and longevity of patients with severe cardiovascular and peripheral vascular disease [134]. A second method involves the modifications of graft surface in order to facilitate the formation of a complete endothelial lining on the graft *in vitro*, prior to implantation [144–147]. Another option is to seed ECs on the luminal side of the graft in an *in vitro* bioreactor, prior to implantation, which involves multisteps processes of harvesting and culturing of autologous ECs or differentiated stem cells derived from the patient. Although *in vivo* studies using EPCs have shown excellent results in terms of graft endothelisation [148–151], very few clinical studies have been undertaken in order to determine its long-term effect on graft patency. Considering the multiple complications associated with re-endothelisation of synthetic materials *in vivo* and the limited availability of stem cell and cell culture laboratories in clinical environments, performing *in vitro* endothelisation before graft implantation is challenging [152].

**Figure 2.**
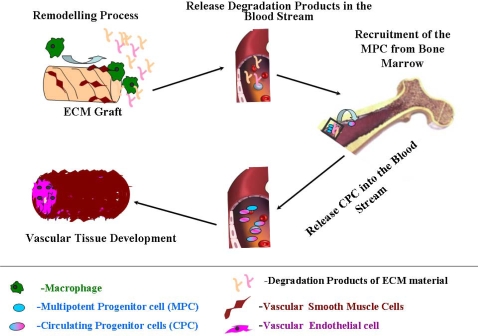
Chemoattractive bioactivity of ECM degradation products. During the ECM remodeling process (due to activity of macrophages, SMC), degradation products are released into the blood stream these recruit multipotent progenitor cells (MPCs) from bone marrow by chemoattractant activity of the peptides and degradation products. The circulating progenitor cells (CPCs) then migrate to the site of implantation and aid in the endothelisation process.

In contrast, ECM grafts show efficient endothelisation *in vivo. In vitro* studies have shown that degradation products of ECM work as chemoattractants which possess chemotactic and/or mitogenic activities and stimulate a migration of fully differentiated mammalian ECs and undifferentiated multipotential progenitor cells to the site from which the product is released [153,154]. Recruitment the patient’s own EPC is presents extremely important beneficial characteristics of this type material and very important for the healing process. Steps which occur during this process are demonstrated in Figure 2.

The effect of incorporation of various biochemical factors (single proteins and peptides) originating from ECM material on synthetic surface in terms of EPC adhesion, growth, migration, differentiation and a graft endothelisation was recently reviewed [155]. The incorporation of single ECM components may present advantages over utilization of the complete matrix alone due to their selective well-defined biological activities and function [156–160]. However, single components of matrix or peptides derived from one type of ECM protein may have a lower activity compared to the native ECM ligand due to the absence of complementary or modulatory domains and amino acids. The process of isolating this component for this use is complex and challenging. Suitable methods which enable the presentation of relatively large biological functional ECM components still have to be determined and optimised.

### 4.2. Bioenergetics of Vascular Healing

As with many other processes of recovery after serious surgical trauma, graft healing and process of new vessel tissue development are very expensive processes in terms of energy consumption [161–163]. It is well recognized that energy is required for open wound healing and a protein deficiency caused by altered nutrient delivery retards healing of the open wound [164–172]. The renewal of the vascular tissue involves several components such as cell proliferation and ECM protein synthesis on the graft site. Both of these components require protein substrates and both metabolic processes are accelerated in order to repair the wound. In addition to that, several energy-consuming biochemical pathways (such as DNA synthesis, maintenance of the membrane potential and intracellular pH level) need to be considered in order to estimate the full energy requirement during this step of graft acceptance. Efficient and rapid delivery of biochemical units (peptides, amino acids) to the site of healing may help sustain the metabolic requirement of the process without need of the additional nutritional diet of the patient or dependance on a fully functioning digestive system (ability to absorb nutrients) and circulatory system (delivery and transport of these nutrients into the site of injury). The biochemical composition and origin of structural groups of synthetic materials are non-comparable to native tissue polymers and as a result have a very low biodegradation rate. Therefore synthetic material does not provide the additional source of energy and material for reconstruction which is required for production of new cells and proteins. The requirement of exogenous energy by the cell, may serve as an important factor influencing neo-tissue development where local limitation in the energy may reduce the replicative capacity of progenitor cells and affect the rate of healing process at the site of the prosthetic vascular grafts. In contrast, ECM material undergoes biodegradation and remodeling which provide energy, biochemical essential structural units and components necessary for protein synthesis and remodeling. All these factors contribute to cell growth, their metabolic activity and development of neo-tissue.

## 5. Vascular tissue Functionality and Homeostasis Maintenance

### 5.1. Biomechanical Properties of Graft Materials and Their Importance in Sufficient Reconstruction of Vascular Tree

Mechanical factors are recognized as important parameters which affect short and long-term graft patencies [4,5,16,173]. Researchers have identified that one of the factors limiting the success of prosthetic vascular grafts is material mismatch with the host vessel in terms of strength, stiffness, compliance and elasticity. Salacinski *et al*., have shown that mechanical properties including compliance mismatch, diameter mismatch and Young’s modulus all greatly affect the graft performance and failure rate [173] and support the idea that an ideal vascular graft must have similar viscoelasticity to the native vessel [174]. Data for compliance and elasticity of native vessels and various small-diameter grafts made from different materials is tabulated in Table 3.

The compliance of 3-layered small diameter SIS grafts was shown to be 4.6% (d = 5 mm) and 8.7% (d = 8 mm) [177], only slightly less than those of carotid and femoral arteries, about four times more compliant than a typical vein graft and an order of magnitude more compliant than modern synthetic vascular grafts of expanded ePTFE and Dacron®. The influence of these particular parameters on flow pattern and hence the cellular healing process will be highlighted in Section 5.2.

Mechanical properties of ECM material vary depending on the animal species, age, organ, physiological and mechanical functions of the organ, size of the organ, species-specific localisation of structural components, protein-homology and protein identity within the tissue and degree of ECM cross-linking [177,182,183]. For example, the modulus of elasticity for canine jejunum is 8.5 MPa [183] compared to that from porcine sources at 7 MPa [177]. Manufacturing and processing parameters such as dehydration time, technique, sterilisation methods and storage conditions are important parameters which could potentially affect the mechanical properties of ECM devices and should be taken into consideration when engineering tissue constructs [182]. The effect of varying the number of layers on the biaxial strength is illustrated in Table 4.

For example, sterilisation by ETO has been shown to have the least detrimental effect upon the mechanical properties of UBM. Gamma and e-beam irradiation decrease the uniaxial and biaxial strength and maximum tangential stiffness. However, ETO had no effect on strength or energy dissipated, indicative of unchanged viscoelasticity. All methods significantly decreased material stiffness, when compared to non-sterilised controls (48–60%) [184].

In addition, the mechanical properties of ECM material are more dynamic than those of synthetic materials due to the susceptibility of ECM material to degradation and remodeling *in vitro* and *in vivo. In vitro* studies have shown that material undergoes ultrastructural changes during incubation with Human Umbilical Vein Endothelial Cells (HUVECs) seeded onto the substrates. UBM weight losses up to 2.5% over a 5 day period were recorded. ECM weight loss correlated to an increased production of metalloproteases MMP-1 and MMP-9, both of which are known contributors to ECM degradation, angiogenesis and vessel remodeling [185]. *In vivo* studies provided complementary results and showed that during graft healing and remodeling, the compliance, modulus of elasticity and burst pressure of ECM graft approached the corresponded mechanical properties of native vessel [186]. Sterilisation of ECM material has been shown to influence on the rate of ECM degradation. An extensive *in vitro* degradation study of SIS over a 49 day period found that e-beam irradiation almost doubled the rate of hydrolytic degradation compared with unsterile SIS, gamma irradiation and ETO (42% versus 23–27%) [187]. These changes are a result of collagen backbone degradation and the difference between the radiation methods could be attributed to the dose and form. Hence, the choice of sterilisation technique should be carefully considered and tailored to the intended application, load bearing requirements and degree of degradability.

In order to engineer a graft which mimics the native soft tissue, manipulation of certain variables such as the origin of the ECM, the number of layers [42,182], the decellularisation method (physical, enzymatic or chemical treatment) [18] and sterilisation techniques (ETO, gamma irradiation, electron beam irradiation) must be determined and elucidiated in order to form a stable reproducable protocol for graft development in each size and substitute location (vein, artery).

The principal strategy being developed to prevent hemodynamic disturbance within the region of the anastomosis is based on the design and fabrication of more compliant ECM–based grafts with viscoelastic properties which mimic those of the human artery. Methods have been introduced to characterize flow structure and wall shear stress (WSS), which may be used in order to provide quantitative comparison of different haemodynamic environments associated with various vascular geometries. Computational fluid dynamics (CFD) and finite element analysis (FEA) softwares may be applied in studies to visualise the flow pattern using velocity vectors, velocity contours and shear stress distribution within the vascular tree or graft replacement region. As these parameters are very difficult to measure *in vivo*, computational modeling can become a nessesary and essential tool of analysis the effect of geometry and shape of the graft placement site on flow pattern and severity of flow alteration [188,189]. Indeed, CFD and FEA offer much more repeatability and resolution than *in vitro* and *in vivo* methods, however, computations must be carefully validated against experimental and clinical data. Preliminary evaluations of graft design have utilised CFD to characterise wall shear stress on tubular ECM grafts and FEA has been used to evaluate stress distributions during mechanical testing of ECM materials; both of these computational methods show good prospects for utilisation in the evaluation of ECM materials as a graft material [190]. The major development of clinical imaging, such as magnetic resonance imaging (MRI) or computed tomography (CT), opens new avenues for detailed patient-specific information on the actual hemodynamics and structural behavior of living tissues. The coupling of CFD/FEA with clinical biomedical imaging technologies may provide an efficient standard evaluation of material performance as a part of clinical practice in the surgical planning and design of graft materials for specific location in the vascular tree.

### 5.2. Mechanotransduction Pathways in the Healing Process of Vascular Graft

It is desirable that the compliance of the graft matches that of the native vessel to avoid potential stagnant regions [181] or disturbances to the local haemodynamics around the anastomosed site. This interruption establishes abnormal pulsatile mechanical stresses at the anastomosis. These stresses can result in suture-line disruption, formation of a false aneurysm, and development of subintimal hyperplasia [3]. These events can either result in thrombosis and increased pannus ingrowth at the anastomosis, thus threatening the patency of the implanted vascular graft. The vascular endothelium is a vital organ, whose healthy physiology and function are essential for normal vascular vessel physiology. The dysfunction of vascular endothelium can be a critical factor in the pathogenesis of vascular disease. ECs lining the blood vessels are transducers of various physiological stimuli which are actively involved in many physiological processes such as regulation of selective permeability, blood coagulation and homing of immune cells to specific sites of the body. Activation of mechanotransductory intracellular pathways is pivotal to shear stress adaptation and is regulated via integrin–mediated connections and cells within the sub-endothelial substrate. These pathways, which are influenced by shear stress, are known to modulate gene expression, cell migration and proliferation. Relationships between hemodynamic parameters [191–199], such as wall shear stress and intra-luminal healing, show that a molecular and cellular cascade is triggered by the various flow patterns created at the anastomosis site which reflects the compliance differences between the material and native vessel. The latter results in turbulent flow, which due to its action on vascular endothelium induces pro-inflammatory and pro-thrombotic expression pathways. Molecular determinants of these cellular pathways stimulated within anastomosis sites of synthetic (PFTE) graft are summarised in Table 5.

As can be seen from the data, molecular determinants of osteogenesisis and vascular bed remodelling are present in the vascular tissue in the area of turbulent flow, where the molecular marker of SMC contractility (like smoothelin) is down regulated demonstrating that blood vessel function and structure are pathologically altered.

It was shown in a previous study by Orr *et al*. [202] that flow-induced activation of the atherogenic transcription factor NF-κβ occurs in a matrix-specific manner. In a study conducted by Jalali *et al*. [203], it was found that EC mechanotransduction in response to shear stresses requires the activation of integrins by their specific ligands which are supported, controlled and influenced by formation of new integrin-ligand connections. Through integrin mechanotransduction, shear stress produced by blood flow upregulates genes involved in regulation of apoptosis, cell cycle arrest, morphological remodelling and nitrogen oxide production; which contribute to atheroprotective effects [204]. Integrins are glycoproteins within the membrane, composed of α and β subunits. To date, 18α and 8β subunits have been identified in mammalian cells [204], with each of these subunits spanning the extracellular and cytoplasmic domain. The major ECM proteins that interact with vascular ECs include collagen, laminin, fibronectin, vitronectin, and fibrinogen [205]. Various ECM proteins have been shown to bind to different integrins, and in turn activate different signalling molecules. For example, collagen type I matrix binds to α2β1 and α1β1 integrins in the endothelium [206–208]. Laminin tends to bind with α6β1 integrin in ECs [209]. Fibronectin mainly binds to α5β1 and αvβ3 integrins. Vitronectin and fibrinogen also bind to αvβ3 integrin [205]. The density and distribution of ECM proteins are known to be controlling factors in the level of integrin-ECM adhesive interaction and play an important role in regulating cell migration [205]. The cytoplasmic domains of both the α and β subunits interact with signalling molecules and cytoskeletal proteins to regulate cellular events (such as signal transduction, cytoskeletal organisation) as well as regulating cell motility via the modulation of integrin affinity and/or avidity.

The integrin-ligand connection acts as a source of communication, transmitting signals from the ECs to the ECM and *vice versa*, coordinating cellular activity. As previously mentioned, the predominant structural component of ECM matrices is collagen; EC attachment to this type of matrix occurs via α2β1 integrins. This interaction inhibits activation and nuclear translocation of NF-κβ and the pro-infammatory molecular cascade under pathological flow conditions. This effect may be beneficial at the early stage of cell repopulation and migration through an anastomosis site, allowing successful tissue reconstruction of the graft without thrombosis developing. Integrin mediated mechanotransduction involves multiple kinases (FAK, c-Src, and Fyn), adaptor molecules (CAS and Shc), guanine nucleotide exchange factors (C3G and SOS) as well as small GTPases (Rap1 and Ras) responsible for activating mitogen-activated protein kinases (MAPKs) such as ERK. In static conditions, the integrins are inactive and signalling does not occur. Shear stress is required to activate the integrins. Through specific interactions of the α and β subunits, the FAK/c-Src and Cav-1/Fyn pathways are activated. Activation pattern is directly associated with members of the Rho small GTPase family, including RhoA, Cdc42, and Rac. RhoA, Cdc42 and Rac each have particular functions in regulating the actin-based cytoskeletal structure. RhoA increases cell contractility, focal adhesions and actin stress fiber formation; Cdc42 regulates filopodia formation; and Rac regulates membrane ruffling [210]. The importance of the β2-integrin family in lipopolysaccharide (LPS) stimulation was highlighted by Monick *et al*. [211]. LPS stimulation plays an important role in regulating the inflammatory process, thus the link between this stimulation and the role of *β2*-integrins is important in terms of tissue remodelling. It has also been found that laminar shear stress suppresses the G1-to S-phase transition in ECs [212,213]. This leads to an increased expression of p21 which inhibits cyclin-dependent kinases, thus inhibiting cell proliferation and remodelling. The regulation of transcription factor expression was compared under disturbed flow conditions and uniform laminar shear stress conditions by Nagel *et al*. [214]. The ECs subjected to disturbed flow, similar to that found in atherosclerosis-prone areas, showed increased levels of nuclear localized NF-κβ, Egr-1, c-Jun and c-Fos compared with those exposed to uniform laminar shear stress or under static conditions. NF-κB induces the transcription of a large range of genes implicated in inflammatory response [215]. It also plays a fundamental role in protecting vascular SMCs against apoptosis and weakening of vascular wall. This transcription factor is stimulated by flow through the integrin and Rac dependent production of reactive oxygen species. It has been previously shown that uniform laminar shear stress plays an inhibitory role in the pro-inflammatory gene expression in ECs located in close proximity to SMC [214,215].

As the specific activation of inflammatory cascades is complex, further studies into the gene expression of various scaffold materials are needed to predict *in vivo* performance. Intuitively it would be expected that the performance of synthetic grafts would be impeded due to their nature and the inability of integrins to bind to corresponding ECM ligands and thus inhibiting of initiation of “healthy” mechanotransduction within the vascular cell. From this point of view, naturally derived scaffolds have a distinct advantage. However, certain ECM components trigger more favourable responses than others. Gene expression in response to physiological fluid flow has yet to be fully characterised for all ECM materials.

**Figure 3.**
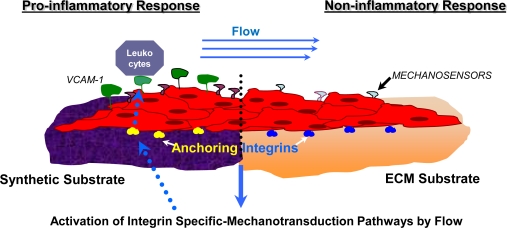
The effect of sub-endothelial substrate on the activation pattern of pro-inflammatory cellular mechanisms of mechanotransduction. This figure illustrates the biochemical processes induced by fluid shear stress for both a synthetic material and ECM scaffold material. As fluid flows across the EC layer (depicted in red), the mechanosensors at the endothelium surface sense the stress and react by transmitting signals through the transmembrane integrins (illustrated in red and blue). Specific integrin mechanotransduction pathways are thus activated, sending messages from ‘inside-out’ through specific integrins, triggering certain responses. Depending on which type of integrin is activated, various responses occur. Synthetic materials are prone to stimulate a pro-inflammatory response of adhered cell [217] and exposed to the flow, in which presentation on the surface of vascular endothelium of adhesion molecules (for example VCAM-1) induce a subsequent attraction, rolling and adhesion and subsequent attachment of leukocytes to the site.

In a study conducted by Cenni *et al*. [216], integrin expression was evaluated for ECs alone and ECs in contact with polyethylene terephthalate (PET) woven Dacron. The following integrins were evaluated under both conditions by flow cytometry: VLA-2 (α2β1-CD49b/CD29), VLA-5 (α5β1-CD49e/CD29), VLA-6 (α6β1-CD49f/CD29) and αVβ3-CD51/CD61). The isolated ECs in contact with woven Dacron showed a significant decrease in the expression of CD29 and CD49e and the other integrins were not modified by contact with the material. CD29 and CD49e are the α5β1 integrin types which are known to have affinity to fibronectin ligands and are important for cell adhesion. The decrease in this integrin type may suggest that adhesion chemistry between vascular cell and synthetic material differs from that of cell adhesion to natural ECM components. Strength of adhesion and retention under the flow shear stress and rate of EC migration on this type of substrate may lead to incomplete endothelial lining which may result in thrombogenicity [216]. To combat these issues, synthetic materials such as PET and PTFE are often coated in fibronectin. Plasma treated PET and PTFE have also shown improved adhesion and growth of ECs [217]. Depending on the nature and origin of ECM materials, different integrins are expressed. Activation of certain integrins suppresses the activation of other specific integrins, maintaining a balance which aims to promote healthy tissue remodelling. Manipulation of integrin activation could eliminate adverse remodelling events, for example, by blocking the inhibition of integrin α2β1 (activated on fibronectin through protein kinase Cα) then collagen signaling can occur via this integrin and inhibit the flow induced activation of the atherogenic transcription factor NF-κβ [218]. The state of EC surface thrombogenicity is under substrate control, and is also related to the cellular differentiation status (as shown in Figure 3).

These cellular processes demonstrate the potential of the underlying vascular material to affect the long-term cellular functionality of the prosthesis. *Ex vivo* evaluation of the material properties in order to support functionality of the EC and their mechanotransduction capacities require optimization. *In vitro* studies under static conditions have been popular for characterization of EC thrombogenicity and shear resistance due to their logistical simplicity, but are not necessarily reflective of the surface thrombogenicity under flow conditions where mechanotransduction activation in a fashion similar to the vascular tree *in vivo* are necessary and its modulation by substrates may be analysed and determined. A biorheological conditioning protocol proposed by O’Keeffe *et al.* [219] can provide a efficient informative *in vitro* screening method which would allow determination of vascular cell behaviour on surface graft material under pathological shear stress, elucidate an alteration pattern of the molecular cascade of endothelial cell mechanotransduction, investigate the ability of materials to support a polarization and EC cytoskeleton reorganisation under various flow patterns (which may be recreated in the anastomosis site of a graft vessel). The undestanding of molecular mechanisms of graft failure will lead to the possibility of further modification of the vascular material in order to enhance clinical performance of prosthetic and ECM grafts.

### 5.3. Restoration of Innervation and Blood Vessel Homeostasis

In recent years, biomedical research has clarified the involvement of neuromodulation in human tissue processes which occur during healing [220,221]. However, limited data exists on the re-innervation pattern of vascular graft materials during the healing process due to the complexity of the neuron detection and analysis [222,223]. However even limited findings have demonstrated the role of innervation and its pattern may play a part in the development of fully functional tissue during the healing of vascular graft [224,225].

**Figure 4.**
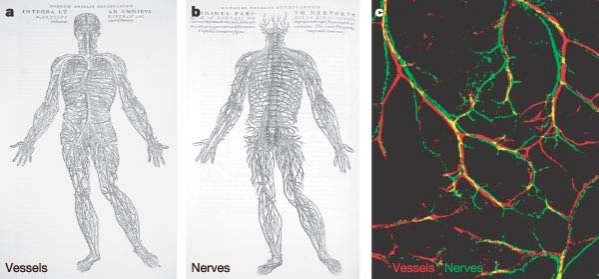
Illustration by the Belgian anatomist Andreas Vesalius, highlighting the similarities in the arborisation of the vascular and nervous networks. Vessels (red) and nerves (green). [227], *Copyright, Reprinted with permission from the Nature Publishing Group.*

Anatomical investigations reveal that blood vessels and nerve fibres run throughout the body alongside one another and the mechanisms involved in wiring both networks are proposed to be similar (Figure 4) [226–228]. Recent morphological and pharmacological findings support the hypothesis of active communication between vascular and neural networks and their interactions may contribute to the health and homeostasis of vascular vessels [229–231].

The blood vessels are innervated by the autonomic nervous system. Sympathetic adrenergic nerves, which travel along arteries and nerves, are found in the adventitia (outer wall of a blood vessel). The sympathetic fibres mediate a vasoconstrictive action in the vascular bed as well as providing secretomotor activity. Activation of vascular sympathetic nerves cause vasoconstriction of arteries and veins mediated by α-adrenoreceptors [232,233]. Neurogenic control of vascular tone and vascular innervations of the blood vessel adventitia have been well documented [230,231]. The release of neurotransmitter and chemical signalling occurs in small enlargements along the nerve fibres. Nerve stimulation can elicit different responses, in terms of type and amplitude, at different areas of the vascular system [234,235]. Neurogenic vasocontrol can thus follow different patterns depending on which molecules are released and what local reactions they trigger. Active molecules released locally from adventitial nerves may diffuse and act directly on the adventitia and the media or act on the endothelium which in turn will release molecular signals (like nitrogen oxide) which then influences the media [229]. The complexity of neurochemically defined autonomic nerves stimulating the vessel baroreceptor and chemoreceptor regions suggests functionally separate, independently regulated pathways. Auger *et al.* [230] summarised previous literature and concluded that vascular autonomic and various types of sensory nerves inclusive of cholinergic, adrenergic, peptidergic or nitrergic are found in different proportions and density depending on the specific anatomical site. One of the findings of this study was that the variety of nerves which can be found in the adventitia is directly related to the presence of many neuron-related peptides and molecules (such as acetylcholine, noradrenaline, neuropeptide Y, substance P(SP), calcitonin gene-related peptide (CGRP), neurotensin and vasoactive intestinal peptides (VIP)) in different quantities at various anatomical regions, although their complete physiological roles are not yet known.

Modern surgical procedures utilized for implantation of vascular grafts have been shown to cause extensive damage to the sympathetic nerves which supply and accompany blood vessels [237,238]. Some procedures may cause extensive degeneration of adrenergic nerves and the extent of denervation may vary with vessel type with regard to their anatomical characteristics and structure (elastic or muscular). Comparative studies were able to determine that rate of nerve re-growth in muscular vessels is faster than that of elastic vessel. Re-growth of injured fibers can be altered and lead to hyper or denervation along the graft reconstruction or only at certain parts. Preliminary analysis of data indicates that a non-matching to the original pattern of nerve re-growth may lead to a lack or alteration of vessel tone autoregulation in the graft region and its exclusion from the baroreflex modulation of blood flow. This growth pattern is potentially regulated by a number of factors such as chemical affinity of the material surface, cytokine and growth factor gradient along the grafted site, the type of ECM molecule deposition, adsorption to the material, the presence and distribution of required chemical ligand to the surface and within the scaffold, inflammatory sites and infection.

In newly developed vascular tissue, neuromodulation activity or its complete absence appears to greatly affect the functionality of the vessel itself, the healing process of the vessel and its homeostasis. Instability, due to focal unbalancing of constrictive forces and regulated, molecular signaling may serve as a pathophysiological basis for the well-established phenomenon of vascular SMC hypertrophy and hyperplasia after grafting [239], occlusion of the vascular graft [240] or its dilation (pseudo aneurysms) [241].

Recent studies have reported successful re-innervation of ECM reconstructed organs [242–244], and the capacity of ECM materials to support nerve conduits and promote growth of the Schwann cell (SC) [244–248]. During biocompatibility studies *in vitro* [248], when co-cultured with SCs, SIS-ECM showed good ability to support SCs adhesion, survival, migration and proliferation on its surface. Observation of the ultrastructure of SCs by TEM demonstrated that SCs adhered tightly and grew productively on the surface of SIS. MTT assay also showed that SIS did not have a cytotoxic effect on SCs. Quantitative analysis of nerve growth factor-β (NGF-β) and brain-derived neurotrophic factor (BDNF) by ELISA, as well as semi-quantitative analysis of NGF-β mRNA and BDNF mRNA by RT–PCR, showed that SCs seeded on SIS had more productive function of secretion than the normal cultured SCs. NGF-β and BDNF are the main growth factors secreted by SCs, which are known to have neurotrophic effects on nerve regeneration. Adhesion and growth of the nerve cells is guided by specific structural components of ECM laminins [249–253]. There is a significant lack of research focusing on the analysis of innervation fibre density, their localization in the adventia and media of the synthetic and ECM grafts. This issue needs to be elucidated and determined in future studies, but the authors hypothesize that ECM graft material may provide a better support for the re-innervation of the newly developed vascular tissue and an enhanced structural and chemical microenvironment for the reconstruction of a balanced interactive network between the nervous system and remodeled vascular tissue. ECM material has the potential to provide a homeostatic environment based on the regulation of vasoactivity function, thus increasing its ability to regulate blood flow and function in a similar manner to the native non-injured vessel compared with vessels substituted by synthetic materials.

## 6. Future Perspectives for Cardiovascular Implants Based on ECM

With the enhanced healing properties previously discussed, biologically derived ECM materials have been shown to be diverse and inconsistent in both structure and morphology [41], and affected by a number of factors such as the manufacturing process (*i.e.*, mechanical decellularisation vs. chemical decellularisation) [18] and the age and health status of the animal at harvest. Numerous limitations and problems are associated with decellularising techniques and procedures, as described previously in detail [18,254–256], may have great influence on ECM product quality, mechanical and biochemical properties, biocompatibility and clinical performance [257–259]. Mechanical methods of acellularization, including repeated freeze-thawing, sonication, or other physical means of disrupting cells’ plasma membranes, provide a direct, mild and rapid tissue decellularisation, but used alone, such methods are not capable of completely removing cellular material, which has been shown to prevent complete recellularisation of this material by host cells [257–259]. It remains a concern for the biomedical community that trace amounts of potentially antigenic compounds of animal origins (lipids, DNA, glycosilation products) have been reported to be present for certain types of ECM material and may provoke an inflammatory response at the placement site [260,261]. Complete removal of these antigenic compounds from the material is important for improving the biocompatibility of ECM material; however, such a goal seems to be quite challenging and difficult to achieve with the application of standard biochemical extraction methods, due to the high degree of chemical complexity and variability of the contaminants. The degradation and damage of structural components due to the absence of a long-active protease inhibitors, multiple incubation and rinsing steps during the long decellularisation procedures may unintentionally remove desirable ECM components and lead to an alteration of mechanical properties of the ECM material [262]. The ability of a decellularisation method to sufficiently remove of lipid moeities from the tissue has been shown to have a dramatic impact on the rate of graft calcification and substantially decrease patency time *in vitro* [263,264]. Minimally invasive tissue-specific decellularisation techniques with detailed manufacturing protocols where *in vivo* performance will be supported by enhanced cell repopulation, minimal infiltration of inflammatory cell and calcification still need to be developed in the near future.

Suitability of materials derived from various organs for clinical application in the vascular area are still under investigation as the biochemical and biophysical properties are not always tailored to the potential tissue characteristics desirable for the application [17,18]. Modification, remodelling and ECM matrix deposition that can be performed *in vitro* utilising cellular machinery able to perform the most complex synthesis reactions and cleavage of others with high specificity and efficiency, presents a potential method to improve the biological properties of ECM material obtained by decellularisation of non-vascular organs (SIS) for vascular application [265]. SIS-ECM material was pre-conditioned with human endothelial cells *in vitro* and during the conditioning process, remodelling of the matrix and synthesis of novel components as well as deposition of sub-endothelial matrix (basement membrane) known to be absent in the original SIS material, was shown to occur. Following decellularisation of the cell-seeded scaffold, neo-ECM was shown to have improved biological activity and the vascular endothelial cells seeded on the neo-matrix had enhanced organization of the cell junction, an increased metabolic activity and released a lower amount of pro-inflammatory prostaglandin PG1 compared to the cells incubated on the control SIS. Neo-ECMs were also shown to have a lower degree of human platelet adhesion and improved thrombogenic potential.

In this state-of-the-art era, with strong development in genetic engineering methods [266] and the multitude of recombinant vectors [267], gene delivery systems [268–280], the variety of cell lines which are able to express recombinant ECM molecules, enzymes and growth factors [281–283] all show great promise for ECM material modification. A more complete understanding has emerged of the native molecular regulation of the ECM remodelling process by the cells under chemical and/or mechanical stimulation (flow [284], stretch [285], pressure [286]), providing an optimised physiological environment within an engineered bioreactor. This may facilitate a more physiological remodelling process, ultimately leading to a more manufacturable tissue-specific ECM material. Advanced bioreactor design and application will be an essential part of conditioning the ECM material and ECM-material based constructs with further advances in the technology making the engineering of more complex tissues and organs (multi-functional, multi-layered, bio-chemical, bio-properties) a reality in the clinical environment.

ECM material has been shown to a very attractive material as a base or one of the structural components for a composite repair material as part of the continuing challenge to find ways to translate the mechanical properties and clinical performance of ECM biomaterials to vascular clinical applications. In order to increase the mechanical strength of ECM, cross-linking structural components with chemicals such as glutaraldehyde, 1-Ethyl-3-[3-dimethylaminopropyl] carbodiimide hydrochloride (EDC or EDAC) and hexamethylene-diisocyanate is applied; however, modification by this type of method has been shown to have a lower degradation rate *in vivo*, promote early calcifications and changes the host tissue response from an anti-inflammatory, constructive remodelling response to a pro-inflammatory, foreign body response.

Decellularised ECM materials have been incorporated with synthetic scaffolds successfully to date through a number of approaches. A multilayered poly(styrene sulfonate)/poly(allylamine hydrochloride) (PSS/PAH) have recently been used as luminal coatings onto human umbilical arteries, demonstrating a high graft patency post 3 months rabbit implantation and restoring initial compliance of the tissue [287]. Stankus *et al*. developed a composite scaffold containing poly (ester urethane) urea elastomer (PEUU) and UBM [288,289]. As the water-soluble electrospun UBM was a fragile and brittle material, it was blended with PEUU and dissolved in hexafluoroisopropanol and then electrospun. The scaffold was also more resistant to degradation compared to electrospun UBM alone and had improved mechanical properties. The scaffold was both strong and distensible with a tensile strength of 4.9 ± 1.6 MPa and a breaking strain of 85 ± 28%, compared to lyophilized sheets of UBM (0.3–0.4 MPa and 47–67% strain, respectively) [288,290]. After 28 days implantation in a rat subcutaneous model, there was an increase in scaffold degradation and cellular infiltration with increasing UBM proportions. *In vitro* seeding with SMCs displayed enhanced adherence and proliferation with increasing UBM proportions. Most likely, this is due to increased cell adhesion sites retained from the biological component (*e.g.*, collagen, fibronectin, laminin) and growth factors, which survived the enzymatic digestion and acidic conditions during initial processing and electrospinning solvent conditions. Despite the fact that the harsh fluoroalcohols typically employed to electrospin collagen/ECM material effectively denature collagen to gelatin [291], with losses of more than 90% of triple-helical structure, the gelatinous structures with retained growth factors do confer an enhanced bioactivity. The synthetic portion comprising of bioresorbable or non-resorbable meshes such as Prolene™, Vicryl™, Mersilene™, PDS II™, Panacryl™, and Monocryl™ can be introduced by preparation of laminated structures of ECM sheets presenting a new method of manufacturing hybrid-ECM material with enhanced properties. Hence, a synthetic scaffold enhanced with an ECM component may possess more consistent mechanical properties, such as failure strengths, compliance and degree of shrinkage [292], whilst eliminating the need to cross-link or laminate the structures. Incorporation of ECM components into the synthetic material adds several desirable characteristics which are absent even from the most advanced textured synthetic scaffolds and forms a ‘smarter’ biomaterial [293], and introduces new functions of the material with near-physiological multifunctionality of the natural ECM, complex signalling, improved control of cell-matrix interactions [294] and cell-specific matrix response.

Specific ECM components have been commonly used (*e.g.*, collagens, fibronectins, laminins) in cell culture for many years and have been shown to have strong effects on cell attachment and growth. The intricate, ordered nature of the ECM, combined with the complex combination of biomolecular cues, is highly difficult to reproduce with synthetic scaffolds. At best, common synthetic ECMs exploit one or two biomolecular classes. A recent study elegantly demonstrated that the tissue-specific matrix components cause significant differences in adhesion efficiencies, growth rates, morphology and phenotypes of skin, muscle and liver cells, suggesting the need for more appropriate, tissue-specific matrices for *in vitro* cell culture [296]. As mentioned previously, the major disadvantage of synthetic vascular materials is their lack of a confluent endothelium and that they are prone to thrombus induction, embolism and occlusion. They are also less durable than autologous material and are associated with poor healing and lack of compliance and often require extensive use of anticoagulant or antithrombotic agents [297]. Therefore, the importance of creating a suitable endothelium on the luminal surface of any diameter synthetic vessel substitute is paramount. Coatings of proteins, decellularised matrices are being pursued to increase the bioactivity of the prostheses in order to render then more suitable for EC seeding. Common approaches to treat vascular graft surfaces include autologous fibrin coating [298,299] or heparin [300,301] and have been met with mixed success. On the whole, these linings do not provide vital vascular functions such as vascular responsiveness or other biological secretory functions seen with normal blood vessels [302]. A mixture of collagen type I, elastin and poly (D, L-lactide-coglycolide) (PLGA) to impart increased mechanical strength was electrospun to form a non-cytoxic tubular construct with similar compliance to native arteries and minimal inflammatory response [303]. Tillman *et al.* electrospun a collagen type I-PCL tubular scaffold, pre-seeded with ECs and SMCs under bioreactor conditioning flow [304]. The grafts used had a caliber of 5 mm and were able to support EC and SMC growth under pulsatile flow conditions. Although smaller diameters are associated with a higher degree of thrombotic occlusion most remained patent at 1 month, even without the presence of anti-thrombogenic ECs. For example, the Hemashield™ vascular graft is a woven double-velour polyester graft impregnated with weakly cross-linked purified bovine collagen, softened by exposure to glycerol. This confers anti-thrombogenicity, an improved healing response and eliminates the need for pre-clotting, which is patient-specific, time-consuming and troublesome.

Replacement of the vessel, in major surgical cases, may serve as a symptomatic treatment of atherosclerotic lesions or aneurisms that have developed in the vessel wall altering its structure and function but does not target the cause of disease known to include molecular and biochemical processes in the blood and the blood vessel wall, and their interface such as deposition of plaques, cholesterol, platelets and other related molecules within the arterial walls. Due to this factor, long–term performance of the ECM material and functionality of the new vessel tissue depends on the origin of the disease, genetic predisposition, specific diet, age, hormone status and many other risk factors. ECM as a protein–based material may be further modified and activated as a potential delivery method of therapeutic agents (drugs, enzymes, inhibitors) which can be released after incorporation of the material into the body. This approach may only provide a short-term treatment due to quick exhaustion of quantity and activity of the loaded functional protein or drug. Incorporation of the specific DNA molecules with coding sequence of the disease-related gene, its uptake by the host cell during repopulation of the ECM vessel following development of the stable/induced subpopulation of the vascular cell may potentially provide a new resistant to arteriosclerosis development vascular tissue due to continuous or inducible synthesis appropriate enzyme, cellular receptor or ECM components coded by delivered DNA molecules.

The future perspectives for the application of ECM technology to vascular applications still have many obstacles to its success and development, with decellularisation, material properties and component modification processing still at the development stages. The advancement of these technologies should ensure a highly improved bioscaffold for the treatment in cardiovascular applications. Furthermore with the application of new research and approaches to ECM materials may allow many of the outlined shortfalls in the current treatment approaches to be eliminated.

## 7. Conclusions

With our expanding knowledge of molecular cascades during natural healing and their interrelationship, it remains very challenging to develop a material which may avoid the natural recognition system of our body. The creation of a prosthetic vascular graft material for clinical use which will be able to achieve an excellent treatment regime and eliminate many of the current complications requires considerable further scientific investigation.

The long-term patency of the vascular graft is a challenging goal for the vascular surgeon. After the early stages of graft acceptance, the degree of functionality of the developing grafted vessel is regulated by physical and biorheological forces (shear stress, wall pressure, particle deposition). Reconstruction of the active dynamic and complex inter-communication with another system of the human body (neural) has influence on modulation of the cellular and molecular events that underlie regulation of vascular tissue adaptation and final healing. The success of a vascular graft is shown to depend upon the intrinsic properties of the graft material and the hemodynamic environment recreated at the grafting site. The degree to which the homeostatic mechanisms are perturbed, the extent of pathophysiologic responses and their resolution are scales of the host reactions to the biomaterial determine the ultimate success of the graft.

Biological material composed of decellularised tissue matrices has been shown to have the potential to stimulate and augment healing processes via multiple biological activities during symptomatic treatment of atherosclerotic lesions or aneurisms. A range of ECMs have been shown to provide an excellent microenvironment for the many processes that occur in the early stages of healing and allow a long-term maintenance of balanced homeostasis and functional neo-tissue development. Applications of this type of biomaterial in clinical use may lead to successful regeneration therapies for the patient. Further development of techniques to improve the degree of biostability, biocompatibility and calcification potential of a decellularized matrices should be considered in future studies.

## Figures and Tables

**Table 1.** A selection of ECM based products is listed below as identified by Badylak *et al*. [17].

**Product Company**		**Material**	**Processing**	**Form**
AlloDerm	Lifecell	Human skin	Natural	Dry sheet
AlloPatch®	Musculoskeletal Transplant Foundation	Human fascia lata	Natural	Dry sheet
Axis™ dermis	Mentor	Human dermis	Natural	Dry sheet
Bard® Dermal Allograft	Bard	Cadaveric human dermis	Natural	Dry sheet
CuffPatch™	Arthrotek	Porcine SIS	Cross-linked	Hydrated sheet
DurADAPT™	Pegasus Biologicals	Horse pericardium	Cross-linked	Dry sheet
Dura-Guard®	Synovis Surgical	Bovine pericardium	Cross-linked	Hydrated sheet
Durasis®	Cook SIS	Porcine SIS	Natural	Dry sheet
Durepair®	TEI Biosciences	Fetal bovine skin	Natural	Dry sheet
FasLata®	Bard	Cadaveric fascia lata	Natural	Dry sheet
Graft Jacket®	Wright Medical Tech	Human skin	Natural	Dry sheet
Oasis®	Healthpoint	Porcine SIS	Natural	Dry sheet
OrthADAPT™	Pegasus Biologicals	Horse pericardium	Cross-linked	Dry sheet
Pelvicol®	Bard	Porcine dermis	Cross-linked	Hydrated sheet
Peri-Guard®	Synovis Surgical	Bovine pericardium	Cross-linked	Dry sheet
Permacol ™	Tissue Science Laboratories	Porcine skin	Cross-linked	Hydrated sheet
PriMatrix™	TEI Biosciences	Fetal bovine skin	Natural	Dry sheet
Restore ™	DePuy	Porcine SIS	Natural	Dry sheet
Stratasis®	Cook SIS	Porcine SIS	Natural	Dry sheet
SurgiMend ™	TEI Biosciences	Fetal bovine skin	Natural	Dry sheet
Surgisis®	Cook SIS	Porcine SIS	Natural	Dry sheet
Suspend ™	Mentor	Human fascia lata	Natural	Dry sheet
TissueMend®	TEI Biosciences	Fetal bovine skin	Natural	Dry sheet
Vascu-Guard®	Synovis Surgical	Bovine pericardium	Cross-linked	Dry sheet
Veritas®	Synovis Surgical	Bovine pericardium	Cross-linked	Hydrated sheet
Xelma ™	Mölnlycke Health Care	ECM protein, PGA, water		Gel
Xenform ™	TEI Biosciences	Fetal bovine skin	Natural	Dry sheet
Zimmer Collagen Patch®	Tissue Science Laboratories	Porcine dermis	Cross-linked	Hydrated sheet

**Table 2.** Additional ECM based products listed below as identified by the authors.

**Product Com**	**pany Material**		**Processing Form**	
Anginera ™(i)	Theregen	Seeded (ii) Dexon or Vicryl		Sheet
Apligraf®	Apligraf	Seeded (iii) Bovine Collagen I	Cross-linked	Fibrous sheet
Biobrane®	Bertek Pharmaceuticals Inc.	Silicone, Porcine Dermal Collagen I coated Nylon	Covalently bonded	Bi-layered sheet
Biostite®	Vebas S.r.l	Collagen I, HA (iv), CS (v)		Powder
Collagraft™	Zimmer	Bovine dermis, HA, TCP(vi)	Cross-linked	Granules
Collapat II®	Biomet	Calf skin collagen, HA	Cross-linked	Sponge
Healos FX®	DePuy Spine, Inc.	Collagen with HA coating	Cross-linked	Fibrous material
Integra®	Integra LifeSciences	Silicone, Collagen I, GAGs(vii)	Cross-linked	Fibrous sheet
OrCel®	Ortec International Inc.	Collagen I	Cross-linked	Sponge/Gel
TransCyte™	Smith & Nephew	Silicone, Seeded Porcine Dermal Collagen coated Nylon		Bi-layered sheet

(i) Anginera™ was formally known as Dermagraft (ii) Allogenic neonatal fibroblasts (iii) Fibroblasts & keratinocytes (iv) Hydroxyapatite (v) Tricalcium Phosphate (vi) Chondroitin Sulphate (vii) Glycosaminoglycans.

**Table 3.** Compliance and Modulus of Elasticity of native blood vessel and various graft materials.

**Vessel type**	**Compliance**	**Modulus of Elasticity (E)**	**Reference**
Carotid (man)	14.7%	0.4 × 106 dynes/cm^2^	[175]
Carotid (man)	-	6.07 × 106 dynes/cm^2^	[176]
Asc. A (man)	-	0.76 × 106 dynes/cm^2^	[176]
SIS, 3-layer (pig)	4.6–8.7%	8.03 × 106 dynes/cm^2^	[177]
Saphenous Vein	1.96–0.64%	5.5 × 106 dynes/cm^2^	[178,179]
Dacron®	0.76%	56.49 × 106 dynes/cm^2^	[178,180]
ePTFE	0.2%	39.07 × 106 dynes/cm^2^	[180,181]

**Table 4.** Biaxial failure load of multi-laminated bioscaffolds [182].

	**SIS UBM**	
2-layer	42 ± 9 N	19 ± 7 N
4-layer	130 ± 29 N	35 ± 2 N
8-layer	325 ± 53 N	

**Table 5.** Regulation of the molecular cascade in the area of turbulent flow.

**Graft Material**	**Location**	**Biological Response in anastomosis site**			**Reference**
		**Methodology**	**Up-Regulated Biomarkers**	**Down-Regulated Biomarkers**	
PTFE	Carotid artery (Dog)	Microarray, RT-PCR and immunohistochemistry.	(α1) collagen -I, (α2) collagen-I, 80K-L protein (MARCKS), osteopontin, NAP-22, VESPR.	Smoothelin-B, tropomyosin 2 (β), calcium/calmodulin-dependent protein kinase II, RBP-MS types 4 and 5, cysteinerich motor neuron 1	[200]
	Aorta (monkey)	Immunohistochemistry	Osteoblast-specific factor-2 (OSF2)/Cbfα1, (α2)collagen-I, (α1)collagen-III, versican, (α3)collagen-VI, (α2)collagen-V, (α1) collagen-V.	SPARClike-1 (SPARCL1)/hevin, RGS5.	[201]
